# The impact of stenting on hemodynamic environment of tortuous coronary artery: results derived from a numerical simulation model

**DOI:** 10.3389/fbioe.2026.1789824

**Published:** 2026-05-11

**Authors:** Yang Li, Runxin Fang, Genshan Ma, Naifeng Liu, Chengxing Shen, Zhiyong Li

**Affiliations:** 1 Department of Cardiology, Zhongda Hospital, Southeast University, Nanjing, Jiangsu, China; 2 Jiangsu Institute of Metrology, Nanjing, China; 3 Department of Cardiology, Shanghai Sixth People’s Hospital Affiliated to Shanghai Jiao Tong University School of Medicine, Shanghai, China; 4 School of Mechanical, Medical and Process Engineering, Queensland University of Technology, Brisbane, QLD, Australia

**Keywords:** coronary tortuosity, hemodynamics, stenosis, stenting, wall shear stress

## Abstract

**Background:**

Stent implantation in vessels with moderate/severe coronary tortuosity is associated with increased rates of target vessel failure due to higher rates of target vessel-related myocardial infarction or ischemia-driven target vessel revascularization. Local wall shear stress (WSS) changes might contribute to this phenomenon. This study investigates the impact of stenting on the hemodynamic environment of tortuous coronary arteries in a numerical simulation model.

**Materials and methods:**

A numerical simulation model was established to explore the characteristics of hemodynamic parameters before and after simulated stent implantation in tortuous coronary vessels. The numerical simulation model is composed of four tortuous arcs. By controlling the curvature of these arcs, three groups with different tortuosity were formed. Four different stenosis degrees (40%, 50%, 60%, and 70%) were formed by changing the diameter of the third arc in each group. Finally, 12 models with different degrees of stenosis and tortuosity were analyzed.

**Results:**

The velocity and WSS were reduced in proportion to increased stenosis after stent implantation in tortuous segments mimicking tortuous coronary vessels with low, medium, and high tortuosity.

**Conclusion:**

Our results indicate that further reduced WSS in tortuous coronary vessels post stenting might lead to increased endothelial dysfunction, vascular inflammation, and neointimal hyperplasia, all of which facilitate the formation of in-stent restenosis, especially in tortuous coronary vessels with severe stenosis.

## Introduction

Coronary stent implantation is an important therapeutic strategy for alleviating myocardial ischemia, but the long-term efficacy could be significantly affected by in-stent restenosis (ISR) (Klein et al., 2023). A previous study showed that the incidence of ISR was even higher post-stent implantation in torturous coronary vessels (Zhang et al., 2018). The local straightened geometry established after stent implantation was likely to generate a high-risk environment of neointimal hyperplasia and subsequent restenosis after stenting portions of the torturous coronary vessels (Liu et al., 2020). The underlying mechanism is not fully understood.

Fluid mechanics changes post stenting might be involved in the pathogenesis of ISR and stent thrombosis (Koskinas et al., 2012; Wei et al., 2021; Deng et al., 2025). Emerging evidence indicated that stent cell geometry could directly regulate endothelial cell behavior post stenting, thereby influencing endothelialization performance (Ng et al., 2025). A previous study showed that the structural and hemodynamic performance of coronary stents could be significantly affected by the interconnector design of the stents (Ghaffari et al., 2025).

It is known that tortuous vessel segments could promote disturbed blood flow and are associated with regions of low wall shear stress (WSS) and ISR (Bai et al., 2025). Such reductions in WSS are well-established promoters of endothelial dysfunction, vascular inflammation, and neointimal hyperplasia, ultimately increasing the likelihood of ISR. In particular, experimental and patient-specific studies have demonstrated that segments within the lowest quartile of WSS exhibit significantly greater neointimal thickening than segments with higher WSS (Wang et al., 2025). Computational fluid dynamics analyses further revealed that stenting might impose alterations in WSS topology, including patterns of convergence and divergence around stent struts, which contribute to both stent thrombosis and ISR (Wentzel et al., 2008; Ng et al., 2017).

Meta-analyses and systematic reviews in vascular biomechanics and interventional cardiology emphasize lesion morphology, including curvature and tortuosity, as key predictors of restenosis, alongside altered hemodynamic indices such as low time-averaged WSS, elevated oscillatory shear index, and prolonged relative residence time (Chen et al., 2024; Weinberg, 2022). In intracranial and peripheral vascular settings, greater vessel tortuosity has been shown to independently increase ISR risk. Studies in intracranial atherosclerotic stenosis patients demonstrated that vessels exhibiting ISR were significantly more tortuous, and smaller reductions in tortuosity post stenting correlated with increased ISR risk (Eun et al., 2024). Taken together, these findings suggest that coronary tortuosity, by exacerbating WSS disturbances, may independently contribute to higher ISR rates, underscoring the importance of meticulous lesion assessment and tailored interventional planning in tortuous anatomies.

Till now, the contribution and impact of previous and existing stenosis in the setting of vessels with various tortuosity in the process of ISR remain largely unknown. This study investigates the impact of stenting on the hemodynamic environment of a numerical simulation model mimicking coronary tortuosity with various degrees of tortuosity and stenosis. Few similar reports are available.

## Materials and methods

### Model generation

**FIGURE 1:**
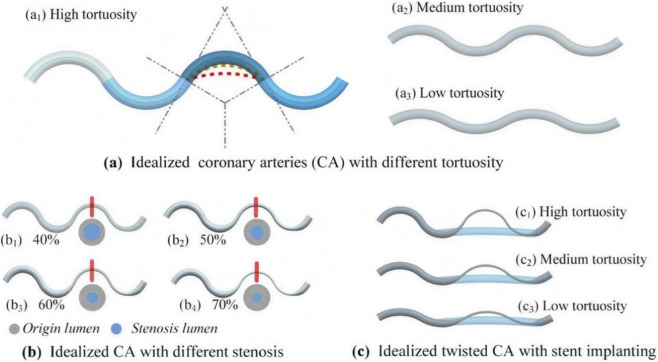
Construction of idealized tortuous coronary artery models. **(a)** Three levels of tortuosity (low, medium, and high) generated by varying arc curvature; **(b)** four stenosis severities (40%, 50%, 60%, and 70%) introduced at the third arc, resulting in 12 models; **(c)** representation of post-stenting geometry by straightening the third arc segment to simulate vessel straightening after stent implantation.

In this study, a basic model mimicking a tortuous artery is composed of four arcs. By controlling the curvature of these arcs, three groups were formed with high, medium, and low tortuosity, as shown in Figure 1a.

By changing the diameter of the third arc in each group, four different stenosis degrees (40%, 50%, 60%, and 70%) were formed. Finally, 12 coronary models with different degrees of stenosis and tortuosity were analyzed (Figure 1b). To study the impact of vessel straightening after stenting, a straightening curve for the third arc in the different models was constructed and analyzed, as shown in Figure 1c.

### Meshing

**FIGURE 2:**
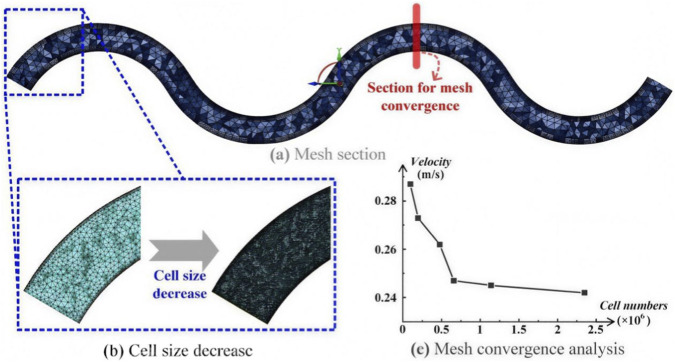
Mesh generation and mesh independence analysis. **(a)** Representative tetrahedral mesh with boundary layer refinement (5 layers). **(b,c)** Mesh convergence study showing variation of average velocity with mesh density. A mesh size of 0.25 mm was selected when the variation was less than 5%.

The ANSYS Fluent Meshing workflow was used to complete the model meshing. Tetrahedral elements were used, and five boundary layers were used to determine the mesh size, as shown in Figure 2. The model was meshed with different cell sizes, and the cell number was increased with the decrease in the mesh size.

Mesh sensitivity was tested by monitoring the average velocity of the stenosed section. A mesh density was accepted when the velocity difference from a denser mesh was less than 5%. The comparison revealed that the average mesh size for this study was 0.25 mm. The models contained 0.6–0.75 million elements. Although WSS was not directly used as the primary indicator for mesh independence assessment, WSS is derived from the near-wall velocity gradient. Therefore, a sufficiently resolved velocity field with appropriate boundary layer refinement is generally considered to provide reliable WSS estimation. In the present study, five boundary layer meshes were applied to improve the accuracy of near-wall flow resolution.

### Computational fluid dynamics analysis

#### Boundary conditions

**FIGURE 3:**
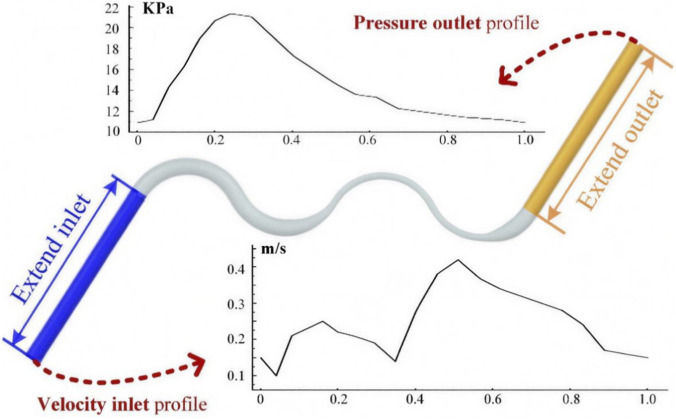
Boundary conditions used in computational fluid dynamics (CFD) simulations. The inlet and outlet were extended to ensure a fully developed flow. A pulsatile velocity waveform was prescribed at the inlet, and a time-dependent pressure condition was applied at the outlet.

Regarding the inlet and outlet settings, the calculation was performed under the transient flow condition. To obtain a fully developed parabolic flow profile, both the inlet and outlet were extended, as shown in Figure 3.

The idealized coronary with an extended inlet and outlet was set to be the velocity-inlet and the pressure-outlet condition. As shown in Figure 3, the time-dependent profiles of the velocity and pressure were given at the inlet and outlet, respectively (Wang et al., 2023; Wang et al., 2020). The inlet velocity waveform used in this study was an idealized pulsatile profile representing a generic cardiac cycle rather than a patient-specific coronary flow measurement. This choice was made to provide a consistent transient loading condition across all idealized geometries and thereby enable a controlled comparison of the relative effects of stenosis and tortuosity.

Regarding the material properties, blood was modeled as an incompressible Newtonian fluid with density 1,060 kg/m^3^ and dynamic viscosity 3.5 mPa s, which are commonly adopted values in cardiovascular CFD studies (Fang et al., 2024; Jiang et al., 2023).

#### Computational settings

In this study, these models were analyzed using ANSYS Fluent (*v* 2021 R1, ANSYS *Inc*., USA). The Semi-Implicit Method for Pressure Linked Equations (SIMPLE) algorithm was utilized to obtain the blood flow velocity, and a pressure-based solver was used for pressure correction and to solve the momentum equation. All the walls are considered rigid and with no-slip conditions. The cardiac cycle was set to 1 s, with a time step of 0.01 s. Two cycles were simulated, considering the instability for the initial time step. Only the final cycle was post-processed for the following analysis.

#### Hemodynamic metrics

The hemodynamic characteristics around the stenosis part of each case were evaluated using two of the most fundamental metrics, velocity magnitude and WSS. Other clinically relevant hemodynamic indices, such as time-averaged wall shear stress (TAWSS), oscillatory shear index (OSI), and relative residence time (RRT) are widely used to characterize disturbed flow patterns. However, in the present study, the analysis focused on velocity and WSS as primary indicators to isolate the effects of stenosis and tortuosity. Future work will incorporate these additional metrics to provide a more comprehensive evaluation of the hemodynamic environment.

**FIGURE 4:**
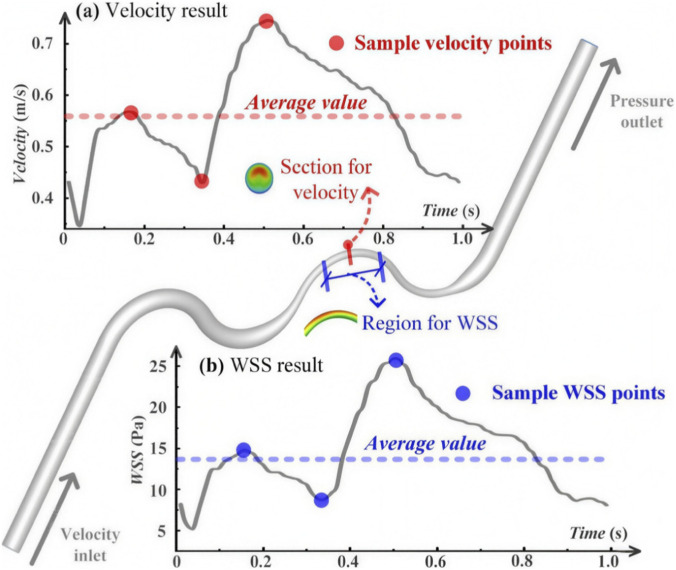
Definition of hemodynamic metrics. **(a)** Velocity magnitude evaluated at three representative time points within the cardiac cycle (selected to capture low, peak, and intermediate flow phases), together with the cycle-averaged value; **(b)** wall shear stress (WSS) evaluated at the same time points.

For the velocity magnitude, as shown in Figures 3, 4a, sample time points and the average velocity value over the cardiac cycle were extracted and compared among the different cases.

WSS measures the viscous stress imposed on the coronary artery walls due to the blood flow. The WSS vector was calculated as follows:

$$\text{wss}=\tau_{t}-\left(\tau_{t}\cdot n\right)n,$$

where $\tau_{t}$ is the wall traction vector calculated from the stress tensor and *n* is the normal direction of the surface. Similarly, the velocity, three sample time points, and the average WSS value over the cardiac cycle were extracted and compared among different cases, as shown in Figure 4b.

## Results

### Velocity reduction

**FIGURE 5:**
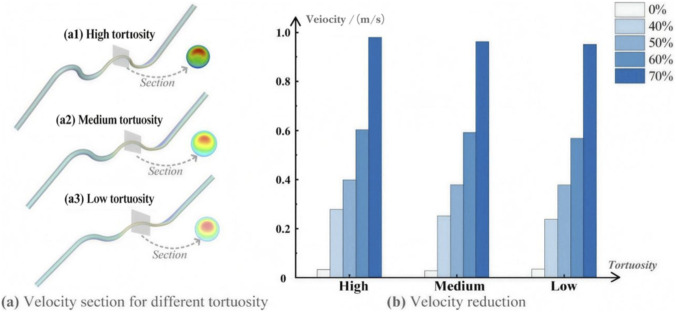
Velocity reduction after stent implantation. **(a)** Intra-group comparison of velocity reduction under different stenosis severities within each tortuosity group. **(a1–a3)** Representative velocity contour distributions (or cross-sectional velocity fields) corresponding to high, medium, and low tortuosity, respectively, illustrating the spatial differences in velocity patterns among different tortuosity conditions. **(b)** Inter-group comparison of velocity reduction among low, medium, and high tortuosity groups at the same stenosis level.

In this work, the reductions in the hemodynamic metrics for these arteries with different stenosis and tortuosity were compared. The details of the velocity data are tabulated in Supplementary Appendix A1. For the three sampled points, the maximum velocity (sample point 3) showed a higher reduction compared with the average velocity, and the velocity reductions for the average value after the stent implantation are shown in Figure 5.

As shown in Figure 5, we first conducted an intra-group comparison. The comparison reveals that within the same range of tortuosity, the higher the degree of coronary artery stenosis, the more pronounced the effect of higher velocity reduction after stent implantation. When performing the inter-group comparisons, we observed that the velocity reduction decreases as the tortuosity decreases. However, this trend is extremely minor compared to the influence of stenosis.

### WSS reduction

**FIGURE 6:**
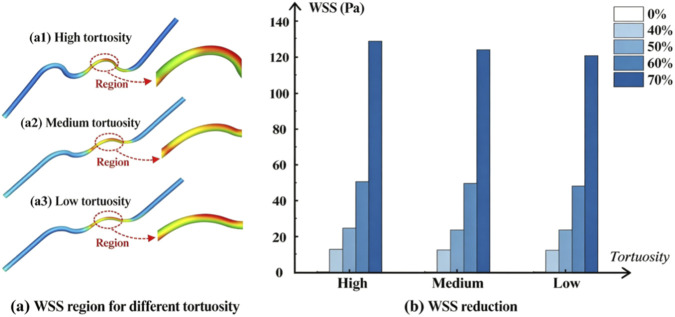
WSS reduction after stent implantation. **(a)** Intra-group comparison of WSS reduction under different stenosis severities within each tortuosity group. **(a1–a3)** Representative WSS distributions corresponding to high, medium, and low tortuosity, respectively, illustrating the spatial differences in wall shear stress patterns among different tortuosity conditions; **(b)** Inter-group comparison of WSS reduction among low, medium, and high tortuosity groups at the same stenosis level.

The analysis of WSS is similar to the velocity-related analysis. For the same tortuosity, the WSS reduction increases with the stenosis increase after stent implantation. When compared under the same stenosis group, the reduction in WSS after stent implantation also shows a positive correlation. However, similar to the velocity analysis, the varying magnitude of this trend is much smaller than the influence of stenosis (Figure 6).

We further examined the percentage reduction after simulated stenting using the average velocity and average WSS values reported in Supplementary Appendix Tables 1, 2. For average velocity, within each tortuosity group, the reduction increased from approximately 41%–44% at 40% stenosis to 73%–74% at 70% stenosis, corresponding to an overall stenosis-related variation of approximately 29–33 percentage points. In contrast, for a fixed stenosis level, the variation attributable to tortuosity was much smaller, ranging from approximately 0.2 to 3.4 percentage points across the three tortuosity groups. For average WSS, the reduction increased from approximately 86.4%–87.0% at 40% stenosis to 98.48%–98.50% at 70% stenosis, yielding a stenosis-related variation of approximately 11.5–12.0 percentage points, whereas the variation across tortuosity groups at a fixed stenosis level was only approximately 0.02–0.53 percentage points.

## Discussion

The main finding of the current study is that the velocity and WSS were significantly reduced in proportion to increased stenosis after stent implantation in tortuous tube segments mimicking tortuous coronary vessels with low, medium, and high tortuosity. Because the reduction of WSS is related to ISR, our results, derived from the numerical simulation model mimicking coronary vessels of tortuosity with different degrees of tortuosity and stenosis, thus provided the experimental evidence referring to the increased risk of ISR post stenting in tortuous vessels. Importantly, the reduction in WSS is more significant in tortuous vessels with severe stenosis. Results from quantitative comparisons also indicate that the magnitude of hemodynamic change is more strongly associated with stenosis severity than with tortuosity. Under the present idealized simulation framework, the magnitude of velocity and WSS reduction varies much more with stenosis severity than with tortuosity, suggesting that stenosis might exert a stronger effect on these hemodynamic indices in this model. To our best knowledge, that is the first report with the numerical simulation model mimicking coronary vessels with different degrees of tortuosity and stenosis. The results derived from this study are based on rigid vessel wall models. Coronary arteries are not rigid and undergo deformation during the cardiac cycle. Future studies are needed to determine the fluid-structure interaction between soft and rigid vessels on wall shear stress and velocity patterns.

Multiple clinical implications of coronary tortuosity have been explored in previous studies. Stent implantation in vessels with moderate/severe coronary tortuosity is associated with increased rates of target vessel failure due to greater rates of target vessel-related myocardial infarction and ischemia-driven target vessel revascularization (Konigstein et al., 2021). Severe coronary tortuosity is the strongest independent predictor of non-obstructive coronary artery disease, followed by female gender and left coronary dominance (Zebic Mihic et al., 2023). A study found that patients with spontaneous coronary artery dissection with coronary artery tortuosity were older, with a higher prevalence of fibromuscular dysplasia (Tweet et al., 2024). Coronary tortuosity is associated with decreased coronary flow reserve and an increased index of microcirculatory resistance (Li et al., 2020).

The present study highlights the need for pre-stenting decision making on tortuous vessels, especially those with severe stenosis, as stenting on these coronary segments might be linked with a significantly increased risk of ISR due to the immense reduction of WSS, a phenomenon associated with endothelial dysfunction, vascular inflammation, and neointimal hyperplasia, all jointly contributing to the formation of ISR. Our study highlights the urgent need for post-stenting medication and monitoring of patients with tortuous coronary vessels with severe stenosis to identify which measures might reduce the incidence of ISR in these high-risk patients.

## Limitations and future prospective

Note that the present study only described post-stenting hemodynamics; the impact of stent geometry was not considered in the computational model. Our study only partly represented vessel straightening rather than true stent implantation. These limitations must be kept in mind when interpreting current results, and future studies are needed to explore related issues by modifying the modeling and observing related WSS changes. The present study relies on an idealized four-arc coronary geometry rather than patient-specific anatomy. The physiological and clinical relevance of the results derived from this simplified model must be further explored. The study represents stent implantation by straightening the vessel segment but does not model actual stent strut geometry. Future studies are warranted to determine how this omission of stent structures might affect the local hemodynamic environment.

To more accurately capture the hemodynamic consequences of coronary tortuosity, future computational studies should consider constructing patient-specific three-dimensional vascular models, incorporating not only vessel geometry but also stretching and deformation of the vascular wall. Moreover, the inclusion of greater vessel curvature and tortuous configurations may better reflect the influence of stent deployment in complex anatomies on WSS distributions.

The hemodynamic assessment in our model relies only on velocity and WSS. Additional clinically relevant hemodynamic indices, including time-averaged wall shear stress (TAWSS), oscillatory shear index (OSI), and relative residence time (RRT), are known to better characterize flow disturbance and endothelial risk. Their absence in the current study represents a limitation, and future work will integrate these parameters to enhance the mechanistic interpretation of coronary hemodynamics. We admit that the computational fluid dynamics model lacks validation through benchmark comparison, experimental reference, or literature-supported verification; this limitation might impact the reliability of the reported hemodynamic findings. Given the simplified geometry and lack of clinical validation, how generalizable the findings are to real-world coronary interventions remains uncertain.

Blood was modeled as a Newtonian fluid in the present study, which is a widely accepted and commonly adopted assumption in cardiovascular CFD and is generally sufficient to capture the dominant flow patterns and global hemodynamic characteristics, particularly under moderate-to-high shear conditions (Saqr et al., 2019). Although blood exhibits non-Newtonian shear-thinning behavior, previous studies have shown that its effects are mainly confined to local regions with low shear rate or flow recirculation, such as recirculation zones or near stenotic/post-stenotic regions, and do not substantially alter the overall flow trends (Morales et al., 2013). Nevertheless, non-Newtonian effects may influence local flow characteristics and the quantitative evaluation of hemodynamic indices such as WSS, TAWSS, OSI, and RRT, especially in disturbed flow regions (Doost et al., 2016). Therefore, while the Newtonian assumption is considered a reasonable simplification for the present idealized comparative study, future work will incorporate non-Newtonian models to further improve the physiological accuracy of the simulations.

Subsequent research may further benefit from comparative analyses based on clinical imaging data, thereby enhancing the translational relevance of computational fluid dynamics simulations to real-world patient outcomes. Finally, mesh independence analysis is insufficient in this study; future studies must show both convergence and velocity changes to accurately present WSS changes.

In conclusion, our results indicate that further reduced WSS in tortuous coronary vessels post stenting might possibly lead to increased endothelial dysfunction, vascular inflammation, and neointimal hyperplasia, all of which facilitate the formation of ISR, especially in tortuous coronary vessels with severe stenosis. Future clinical studies are needed to validate results derived from this numerical simulation model mimicking coronary vessels of tortuosity with different degrees of tortuosity and stenosis.

## Data Availability

The original contributions presented in the study are included in the article/Supplementary Material; further inquiries can be directed to the corresponding authors.
