# Supplementary material for: The impact of stenting on hemodynamic environment of tortuous coronary artery: results derived from a numerical simulation model
Source: Front Bioeng Biotechnol. 2026 May 11;14:1789824. doi: 10.3389/fbioe.2026.1789824 (PMC13199118; doi:10.3389/fbioe.2026.1789824)
Supplement: Supplementary file 1 [file Supplementaryfile1.docx]

**Appendix**

Table 1 Velocity value

| Tortuosity | Value | Stenosis | | | | | Stenting |
| --- | --- | --- | --- | --- | --- | --- | --- |
|  |  | 0% | 40% | 50% | 60% | 70% |  |
| High | Average | 0.3820 | 0.6282 | 0.7488 | 0.9531 | 1.3289 | 0.3495 |
|  | Sample 1 | 0.3825 | 0.6243 | 0.7437 | 0.9473 | 1.3218 | 0.3479 |
|  | Sample 2 | 0.3066 | 0.5041 | 0.5974 | 0.7537 | 1.0513 | 0.2799 |
|  | Sample 3 | 0.5000 | 0.8215 | 0.9796 | 1.2491 | 1.7502 | 0.4563 |
| Medium | Average | 0.3770 | 0.5997 | 0.7276 | 0.9408 | 1.3110 | 0.3480 |
|  | Sample 1 | 0.3774 | 0.5979 | 0.7208 | 0.9293 | 1.3039 | 0.3511 |
|  | Sample 2 | 0.3024 | 0.4780 | 0.5794 | 0.7543 | 1.0428 | 0.2784 |
|  | Sample 3 | 0.4928 | 0.7843 | 0.9506 | 1.2316 | 1.7274 | 0.4567 |
| Low | Average | 0.3775 | 0.5800 | 0.7204 | 0.9103 | 1.2949 | 0.3424 |
|  | Sample 1 | 0.3768 | 0.5782 | 0.7179 | 0.9029 | 1.2867 | 0.3440 |
|  | Sample 2 | 0.3025 | 0.4616 | 0.5737 | 0.7255 | 1.0307 | 0.2750 |
|  | Sample 3 | 0.4935 | 0.7588 | 0.9449 | 1.1966 | 1.7031 | 0.4472 |

Table 2 WSS value

| Tortuosity | Value | Stenosis | | | | | Stenting |
| --- | --- | --- | --- | --- | --- | --- | --- |
|  |  | 0% | 40% | 50% | 60% | 70% |  |
| High | Average | 2.5804 | 14.7096 | 26.4825 | 52.5013 | 130.9256 | 1.9951 |
|  | Sample 1 | 2.8668 | 14.5551 | 25.7276 | 50.2428 | 122.8410 | 2.0592 |
|  | Sample 2 | 1.6857 | 9.0894 | 15.8644 | 30.6321 | 75.8186 | 1.2077 |
|  | Sample 3 | 4.5126 | 25.5661 | 45.2221 | 89.8531 | 231.2304 | 3.5495 |
| Medium | Average | 2.4923 | 14.3957 | 25.5717 | 51.4358 | 126.0420 | 1.8932 |
|  | Sample 1 | 2.7840 | 14.0491 | 24.6632 | 49.1054 | 119.3287 | 2.1036 |
|  | Sample 2 | 1.6276 | 8.7103 | 15.3216 | 30.7609 | 74.4377 | 1.1321 |
|  | Sample 3 | 4.2615 | 25.1224 | 44.0804 | 88.8055 | 220.7853 | 3.6506 |
| Low | Average | 2.4451 | 14.1253 | 25.3745 | 49.8646 | 122.5383 | 1.8406 |
|  | Sample 1 | 2.7026 | 13.7743 | 24.4320 | 47.6428 | 116.1398 | 2.0000 |
|  | Sample 2 | 1.5908 | 8.4807 | 15.1118 | 29.6622 | 72.1988 | 1.1449 |
|  | Sample 3 | 4.2127 | 24.1207 | 43.5052 | 86.0903 | 213.5186 | 3.3253 |
